# Need, Strategies and Requirements in the Medical System for Bone Banks: A Review Article

**DOI:** 10.7759/cureus.28785

**Published:** 2022-09-05

**Authors:** Priyanshu R Verma, Ashish Anjankar, Parth V Singh

**Affiliations:** 1 Anatomy, Jawaharlal Nehru Medical College, Datta Meghe Institute of Medical Sciences, Wardha, IND; 2 Biochemistry, Jawaharlal Nehru Medical College, Datta Meghe Institute of Medical Sciences, Wardha, IND; 3 Internal Medicine, Indira Gandhi Government Medical College, Nagpur, IND

**Keywords:** grafting, preserving, donors, guidelines, bone bank

## Abstract

An orthopedic bone bank's creation and management is a challenging procedure where medical organization and legal requirements interact. There are no formal regulations for the management and organization of an orthopedic bone bank in the Netherlands or any other nation in Europe. The recently revised "law of security and quality for utilizing human materials in the Netherlands establishes guidelines for the technical and administrative elements of using human tissue and cells. The bone bank's processes involve a rigorous questionnaire for choosing donors, a complete bacteriological, histological, and serological examination, as well as industry-standard, practices for registering, processing, preserving, distributing, and storing bone allografts. This article explains how an approved bone bank is run, and it may be used as a suggestion for formal regulation or as a model for additional orthopedic bone banks in Europe. Osseous graft manufacture, testing, packing, storage, and transportation are all handled by bone banks. Their primary responsibility is to guarantee the transplants' biological characteristics and microbial cleanliness by legal and quality criteria. All orthopedic surgeons face the challenge of reconstructing bone defects; to address this issue, there are several methods, including the use of autografts, allografts, and bone substitutes to enhance and speed bone recovery. Although autografts have superior biological qualities, their volume is constrained and they are linked to donor site morbidity. Allografts are readily accessible, however, there are still worries about the possibility of infections, and they lack osteosarcoma qualities.

## Introduction and background

In today’s world of bone grafting, it is necessary to have bone stored so that we could look for bone grafts easily. Donor bone from the orthopedic bank is frequently required for the reconstruction of bone defects [1]. The clinical situation and the desired outcome will determine the type of bone transplant that is utilized [2]. Bone transplants are used to repair abnormalities caused by trauma, infection, and the removal of bone tumors [3], in the fusion of the spine [4], and as impaction grafts in total joint arthroplasty revision [5]. With longer life expectancies, osteoarthritis is one of the most common chronic illnesses today, and both its frequency and occurrence are predicted to grow. With significant costs to society and health care, this disorder is chronic and results in functional decline and the potential for loss [6]. Arthroplasty continues to be the sole available end-point therapy. Age might be the main source of risk for osteoarthritis [7].

Although autologous bone can start new development and can grow on the surface of the bone, it is typically not available at the desired spot [8]. Only allogenic bone can develop on the surface of the bone; it acts as a framework for freshly created bone from which it may begin to generate [9]. The inflammation, which results in the production of many cytokines and growth factors, is what starts osteogenesis. It causes stem cells to be drawn in and new blood vessels to develop, which results in the development of functional bone [10].

The VU University Medical Center (VUmc) in Amsterdam, which houses femoral heads for appropriate patients who had undergone hip replacement surgery, is one example of a hospital operating its bone bank. When a patient needs bone surgery, bone grafting surgery, or bone replacement surgery, it has the advantage that they sometimes do not have to hunt for bone donors. Additionally, it is advantageous financially for the associated hospitals. In India, bone banks may be found in institutions like Parvathy Ortho Hospital GST Road, Chrompet, Chennai, M/s Ganga Medical Centre & Hospitals Private Limited, Mettupalayam Road, Coimbatore 641043, Rajiv Gandhi Government General Hospital, and Cancer Institute, WIA [11].

Dutch Bone Bank Foundation (NBF) was established in the city of Leiden, Netherlands, in 1988 [12]. The bone and tendon transplant materials from patients who were dead donors are kept in this central bone bank [13]. Hospitals can order such materials from the NBF as needed.

There are currently no nationally recognized standards for the upkeep and administration of bone banks in the Netherlands. The VUmc bone bank technique which uses received official permission, and recognition and may serve as a model for many other institutions is described in this study [14].

## Review

Bank procedures

The orthopedic bone bank of VU University has been recognized officially by the Ministry of Health, Sport, and Welfare as of October 2008. Bank policies are altered to comply with the laws of quality and security for employing materials of the human body [15]. The Netherlands' implementation of this law and the resulting European Directive 2004/23/EC began in the middle of 2007. The technological requirements for processing, coding, presenting, storing, and distributing human tissues and cells are included in these guidelines, together with information on how to report such acts.

In the past, the Association of Musculoskeletal Transplantation of Europe's recommendations were followed. The present organization has ceased to exist as a European umbrella organization due to divergent European laws; at this time, only national associations are in use. Since there is currently no such organization in the Netherlands, there is no national standard for the upkeep and administration of an orthopedic bone bank [16].

The Association of Tissue Bank of America rules is now used in the Netherlands for orthopedic bone banks, and the Council for the Blood Transfusion of the Netherland Red Cross provides requirements for the banks [17], in addition to the recently amalgamated Bio Implant Services and Netherlands Bone Bank Foundation [18].

German musculoskeletal tissue banks are now in a radically different condition as a result of the new tissue legislation of 2007. The new rules in the most current German tissue legislation are intended to increase safety by lowering the danger of spreading viral and nonviral illnesses [19]. In India too still no clear guidelines are available for the same and this article may help in paving a path for the same too.

Bone bank protocols

**Figure 1:**
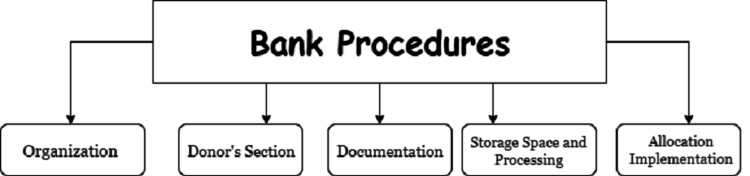
Various Selections in a Bone Bank

An elaborate protocol with the following five sections should be used to properly outline the bone bank procedure: organization, donor selection, documentation, storage place, processing, and allocation implementation. This protocol was created by the administrator of the bone bank and the Head of Department (HOD), of the Orthopedics Division (Figure 1) [20].

Organizations

An organizational chart lists the tasks of many stakeholders, including the head of the department of orthopedics, the bone bank administrator, the theatre nurse, the medical microbiology, an anatomic pathologist, the clinical chemical analyst, the hematological laboratory technician, and the trainer. The administrator is in charge of daily operations, with the HOD serving as the bone bank's chief management. The knowledge and abilities of surgical techniques and clinical cleanliness must be upheld, and this responsibility falls on the orthopedic surgeon and theatre nurse. The administrators of the bone bank are responsible for administration, donor bone storage, and allocation. Additionally, the administrator makes sure that the storage areas are kept clean and well-maintained. The administrator also verifies that the registration papers for femoral heads submitted meet the requirements. The administrator is also responsible for ensuring that the registration documentation for femoral heads that meet the requirements for storage in the bone bank is accurate, as well as maintaining and cleaning the storage facilities (freezers, etc.). Both the trainer and the bone bank administrator are in charge of employee training [21].

Donor Selection

The attending orthopedic surgeon asks the patient for his consent to keep any extracted tissue for donation before performing the hip replacement surgery. It concerns patients whose femoral head grafts will be removed so that a complete hip prosthesis can take their place. Patients having knee or shoulder arthroplasty cannot be considered for bone tissue donation because corticospongious bone tissue cannot be collected in significant quantities.

Documentations

A well-operating bone bank must have accurate paperwork and coding. Each femoral head is given a special registration code. Based on this code, only the administrator of the bone bank may identify the donor. A file is maintained for each registered femoral head that includes the consent documents as well as the outcomes of the bacteriological, histological, and erythrocyte sedimentation rate (ESR) tests. This file also records and stores additional pertinent information, such as the allocation date and the size of the head of the femur. Both the bone bank administrator and the appropriate orthopedic surgeon sign the paperwork after the file is finished, which takes at least six months before the serological examination and there aren’t any problems to be noted. It is now possible to transplant the head of the femur. The head of the femur shall be destroyed in the accordance with hospital practice in the event that a file cannot be completed completely or any aberrant data are reported.

Storage Place and Processing

Surgery is performed to remove the head of the femur under sterile circumstances. Aerobic and anaerobic microorganisms are used to cultivate the ligament and synovial tissue. A biopsy of corticospongious ligaments and bones is taken for histological analysis in order to rule out infections, auto-immune conditions, and cancers. The head of the femur is measured, wrapped in a sterile plastic bag, packaged in three layers with sterile packing material, labeled, and put in the freezer within 30 minutes. A continuous temperature registration device is put in the freezer, which is set at -80°C. A 24-hour guarantee against temperature-related tissue damage is provided by an alarm system that sends a warning signal to the Technical Service if the temperature veers outside the permitted range of -90℃ and -70°C. In the event that the freezer's mechanical components fail, a nitrogen tank is mounted to it as a backup cooling system. When it is extremely frozen, the allogenic bone tissue can only be stored for a maximum of five years. The temperature data is managed and kept for a minimum of five years by bone bank management.

Allocation and Implementation

Femoral heads are removed from the freezer and given to the orthopedic surgeon and surgical team if a surgeon wishes to utilize one as an allograft during surgery. The head of the femur file and expiry date are checked by the orthopedic surgeon and the operating room nurse. The theatre nurse collects a bacterial culture swab after the head of the femur has been defrosted and thawed in physiological saline. The hospital or care institution guarantees that the identifiable criteria are met, which includes preserving the femoral head file and receiving patient information for 30 years after implantation.

Discussion

In 1881, Macewen wrote the first description of the utilization of allogeneic human bone tissue [22]. Allogenic bone transplantation has been used more often since that time and is now a common orthopedic treatment [23]. But during the past 10 years, a lot has changed. Now, precise guidelines must be followed for donor screening, clinical cleanliness, storage and processing, allocation, implantation, and documentation.

Bone allografts facilitate bone reconstruction in orthopedic surgery. Appropriate donor selection and processing of grafts minimize the risk of disease transfer [24]. A comprehensive general survey and an additional physical test are used to choose donors. The survey should be updated on a regular basis to reflect the most recent findings and advancements as well as to address the emergence of new infectious illnesses. For instance, after the severe acute respiratory syndrome coronavirus 2 (SARS-CoV-2) pandemic broke out in a few countries, a question was added to the survey asking potential donors if they had visited areas with the disease or had contact with an infected individual. It is not improbable that recently discovered infectious disorders would be taken into account in the survey and afterward used as exclusion criteria.

Before surgery, a laboratory evaluation that includes ESR measurement is carried out. Elevated levels are often observed with no clinical repercussions [25]. The exclusion standards are closely adhered to, nevertheless. The Rh-factor and blood type are identified before surgery. The Rh-factor is just a concern when a young woman is a recipient patient [26]. In addition, the donor undergoes a serological examination to rule out the spread of illnesses like HIV and hepatitis [27]. Unless expressly requested by the donor, the results are often not disclosed to the donor [28].

In addition to currently existing national and international rules and processes of the American Association of Tissue Bank (AATB) and earlier European Association of Musculo-Skeletal Transplantation (EAMST), histopathological examination is added to the current bone bank approach. It is advised that a histological evaluation of this frequently utilized source of bone allograft be included as part of the screening strategy for bone bank collection given our results that concealed pathological diseases are frequent [29]. Following the recommendations of the Tissue Bank Association and the Association of Musculo-Skeletal Transplantation of Europe, we histopathologically screened all heads of femur extracted during primary total hip replacement from November 1994 through December 2005 and included them in the process for bone banking [30]. Previous investigations have detected pathological anomalies in 8%, and B-cell lymphomas in 2.2% of donor femoral heads [31].

Control of the Local Bone Bank When Japan Declared a State of Emergency Due to COVID-19

Biological allografts for a variety of orthopedic surgeries must be provided by bone banks. Health-care practitioners, institutions, and patients are particularly concerned about how the COVID-19 disease pandemic could affect organ donation and transplantation as countries deal with new realities brought on by the epidemic. Now let us discuss how the Kitasato University Bone Bank was running while a state of emergency was imposed as a result of COVID-19. Allograft bone from donors that tested COVID-19-negative underwent pre-operative polymerase chain reaction (PCR) screening for living donors, and these tissues were then cryopreserved for transplantation.

From February 2-9 to April 5-11, the weekly infection rate steadily climbed in the area where the bank harvests deceased donor-derived allograft bone. It is now known that asymptomatic individuals may transfer the virus, and that this method may have aided in the spread of COVID-19. As a result, the bank stopped accepting donations from deceased donors in order to protect medical personnel. Following bone allograft, none of the patients had any symptoms [32]. The same requires to be followed in our country if such a pandemic strikes in our part of the country too.

Moroccan Authorities Have Established a Bone Bank in the Vicinity of Rabat and Casablanca

Bone grafting has long been used in orthopedic surgery and traumatology, demonstrating its value in these fields. Autografts, which are still used rather regularly, have many issues. Finding the right quality and enough amount of bone is important on the one hand. However, removing the graft adds time to the procedure and is often uncomfortable thereafter. Bone allografts have emerged as a result of the drawbacks of autografts. It is feasible to overcome the different issues presented by bone autografts due to the low immunogenic power of the bone, the graft's good integration, and the simplicity of bone preservation procedures. The necessity for a framework that allows for the administration of graft stocks has arisen as a result of the growing usage of bone allografts. The goal of this paper is to show how a bone bank operates, specifically how it conserves femoral heads without using a further sterilizing procedure or freezing them. The bank first works with all orthopedic physicians in Rabat and Casablanca before expanding to all Moroccan orthopedic surgeons. It offers allografts that are high-quality and secure [33]. This sincerity and template are required for setting a bone bank in our country too.

Upbringing of banks

The bone bank unit that is the focus of this article was created in a teaching hospital for tertiary care in north India in January 2018. This article's goal is to describe where allografts were obtained, how often they were discarded, and how often patients were infected after receiving them.

From extraction and storage through utilization, we outline the Bone and Soft Tissue Bank's methods. Cortical and spongy bone grafts can be utilized in replacement surgery, big post-traumatic reconstructions, and surgeries to rescue cancer patients with significant bone deficiencies when structural support is needed. The former is typically employed in these situations. Due to their many applications, spongy grafts are the most popular. They are particularly helpful in filling holes that need a substantial amount of graft when the autograft is insufficient or as a compliment. Additionally, they are especially beneficial in healing fractures caused by bone loss, delays in fusion, and pseudo-arthrosis in zones with limited blood supply and necrotic tissue. Additionally, they are employed in prosthetic surgery to combat abnormalities of the cavity kind. Soft tissue allografts are particularly recognized in numerous ligament injuries that need repair. Although they may be used to fill any ligament or tendon deficiency, the ones most frequently utilized now are those used in the surgery of the patella. Given that these patients are immunosuppressed from the treatment, the main challenges of cortical allografts are the combination of the ends with the bone itself and in tumor surgery, where the incidence of infection is increased in comparison to spongy grafts and soft tissues, which is irrelevant [34].

Material and method of banks

In musculoskeletal surgery, bone transplants are frequently used to restore mechanical integrity where there is a deficiency and to enable skeletal reconstruction. Auto and allografts have traditionally been utilized. For vast, intricate flaws, the latter is the preferred option. They are available from both live and dead donors. They are kept in facilities known as bone banks using lyophilization and cryopreservation. Before receiving the grafts, donors had their information thoroughly reviewed for common specific criteria, and all relevant data was retained on file. Before storage and immediately before usage, aerobic culture was conducted. Samples with incomplete donor screening, inadequate paperwork, or positive cultures were thrown out [35].

The processes underlying this phenomenon as well as the many aspects affecting osteoinductivity by biomaterials are also described, demonstrating that it is fairly restricted in comparison to osteoinductivity demonstrated by bone morphogenetic proteins (BMPs). As a result, a new word is suggested to define osteoinductivity by biomaterials. Also mentioned are several methods for incorporating osteoinductivity (BMPs, stem cells) into artificial bone replacements [36].

Results

Over two years, the bone bank received allografts from 196 different donors. The head of the femur from complete or partial hip replacements was the main supply of bone. It was necessary to reject 44 (22.4%) grafts. Eighty-eight patients received 95 allografts throughout this period. Bone tumor surgery (40%) was the most frequent reason for usage, followed by difficult initial or revision arthroplasty (30.5%). Following surgery, three patients (3.4%) had a profound infection.

## Conclusions

An orthopedic bone bank's establishment and maintenance as an organ bank is a very complicated procedure where elements of legal structure and medical organization interact. In this paper, the outline of the bone bank technique, that had been authorized and acknowledged by the Ministry of Welfare, Sport and Health of the Dutch Government. The construction and upkeep of other orthopedic bone banks might be accomplished using this technique as a preliminary Dutch standard. In India, we have four bone banks to be precise but this is not enough looking at future needs. Getting a graft from one's own body could be an alternative to the bone bank but it is difficult to have two incisions at a time and go through that pain. In India, we should make protocols taking references from different countries to increase the number of bone banks.
